# The translational potential of inflammation-induced skin blister human models in exploring the pathogenesis of periodontitis and its systemic health implications

**DOI:** 10.3389/fimmu.2024.1469828

**Published:** 2024-12-16

**Authors:** Rizky Aditya Irwandi, Crystal Marruganti, George Collins, Jhonatan de Souza Carvalho, Derek Gilroy, Francesco D’Aiuto

**Affiliations:** ^1^ Periodontology Unit, UCL Eastman Dental Institute, University College London, London, United Kingdom; ^2^ Department of Oral Biology, Faculty of Dentistry, Universitas Indonesia, Jakarta, Indonesia; ^3^ Unit of Periodontology, Endodontology and Restorative Dentistry, Department of Medical Biotechnologies, University of Siena, Siena, Italy; ^4^ Department of Ageing, Rheumatology and Regenerative Medicine, Division of Medicine, University College London, London, United Kingdom; ^5^ Department of Cardiology, St Bartholomew’s Hospital, Barts Health NHS Trust, London, United Kingdom; ^6^ Department of Diagnosis and Surgery, São Paulo State University (UNESP), School of Dentistry, Araraquara, Brazil

**Keywords:** human challenge model, periodontitis, *Porphyromonas gingivalis*, skin blister model, periodontal disease, self-resolving inflammation, periodontitis pathogenesis, periodontitis-systemic link

## Abstract

Periodontitis is a highly prevalent chronic disease. Despite decades of extensive research on the topic, a complete understanding of its immunopathogenesis, especially when linked to other inflammatory comorbidities, is lacking. *Ex vivo* human and *in vivo* animal experiments have shown the host inflammatory response’s crucial role in both the disease’s onset and its systemic implications. These approaches, however, remain questionable when translating these findings into real-world scenarios linked to periodontitis. A clear need for new *in vivo* human models is discussed, especially within the context of understanding the host response to key pathogens linked to periodontitis, such as *Porphyromonas gingivalis* (*P. gingivalis*). Therefore, a skin blister model was employed to describe the stages of the host immune response in humans after challenges by microbial and/or sterile insults. A novel human challenge model using UV-killed *P. gingivalis* holds promise in producing new evidence and bridging the gap of the host response to periodontitis and its links with other common chronic diseases.

## 1 Introduction

Periodontitis is a chronic inflammatory disease characterized by an altered homeostasis between the subgingival microbiome and the host gingival inflammatory response in susceptible individuals (1). A recent report from the World Health Organization (WHO) estimates that the severe form of the disease has affected more than a billion cases worldwide and up to 1/5 of the global adult population (2). Periodontitis is characterized by the progressive destruction of hard and soft tissues supporting the tooth (gingiva, periodontal ligament, and alveolar bone) (3). If left untreated, it will inevitably result in tooth loss, masticatory impairment, and a severe impact on individuals’ quality of life (4). The global burden of this disease across the United States and Europe is estimated to be over $150 billion (5). Its treatment relies upon self-performed improved dental hygiene practices as well as professional dental biofilm removal, with a small minority of patients requiring more sophisticated procedures aimed either at resolving resistant gingival inflammation (localized gum surgeries) and/or attempting to rebuild in part the lost hard and soft tissues supporting the dentition (6). The relevance of periodontitis as a global health problem has reached greater attention over the last 30 years, confirming firm links with a substantial host response characterized by a sustained low-grade systemic inflammation (7) and an altered circulating inflammatory cell profile (8). Over decades, studies have reported that periodontitis is independently associated with numerous inflammatory disorders, such as cardiovascular disease, type-2 diabetes, and rheumatoid arthritis, amongst others (9).

For decades, researchers have tried to understand the pathogenesis of periodontitis, and its possible causal association with other common inflammatory comorbidities has been heavily researched. The latest efforts have focused on the host-pathogens interplay using genetic susceptibility and single-cell transcriptomic analyses, revealing that polymorphisms of inflammatory response genes (10) and innate immune cell dysfunction (11) confer susceptibility of the host to periodontitis. Despite periodontal tissue inflammation resolution following the periodontitis management, the augmented level of some systemic inflammation markers persists, and circulating inflammatory cells still retain their altered periodontitis-associated phenotypes, including increased counts of peripheral blood-derived immune cells (12) demonstrating enhanced inflammatory responses (13, 14). Collectively, the evidence highlights the crucial role of host inflammatory responses (immune cells and their inflammatory mediators) to periodontitis and the underappreciated potential contribution of host immunoinflammatory status to periodontitis-associated systemic inflammation.

To date, studies exploring human immune responses to a keystone pathogen *Porphyromonas gingivalis* (*P. gingivalis*) focus on *ex vivo* human models using tissue cultures (15–19) and local gingival tissue phenotyping (20, 21). Furthermore, animal models of periodontitis have been used to describe and understand the gingival inflammatory response to the pathogen in rodents (22, 23). However, both approaches have numerous limitations, notably that they might not translate into the complexity of the *in vivo* human host response to *P. gingivalis* due to environmental restrictions in a controlled niche of tissue cultures and different animal immune systems. For instance, neutrophil extracellular trap (NET) formation (NETosis) potentiates periodontal inflammation in mice during experimental periodontitis (24). NETosis is evident in periodontal lesions in mice and NET removal by systemic delivery of DNAse-I protects mice from *in vivo* periodontal bone loss. Mechanistically, NETosis is regulated by peptidylarginine deiminase-4 (PAD4)-mediated histone citrullination and this further confirms the role of NETosis in periodontitis – PAD4 KO mice displayed significant protection from periodontal bone loss compared to wild-type (WT) counterparts and *in vivo* pharmacologic delivery of CI-amidine to inhibit PAD4-mediated citrullination protected WT mice from ligature-induced periodontitis (25). However, the mechanism of NETosis in mice is different from that in humans, as experiments of human neutrophils have shown that the cells were still capable of performing NETosis following the PAD4 inhibitor, Cl-amidine (26). In terms of DNAse-I treatment, it failed to degrade NETs purified from neutrophils in humans (27). This conflicting evidence highlights that the mechanism shown in mice was not confirmed in humans. Therefore, the generation of new human *in vivo* evidence is crucial in unveiling the immunological mechanisms of periodontitis and its association with inflammatory comorbidities in humans.

The skin blister model is a recognized *in vivo* human inflammation model that is safely and effectively used to study host-pathogen interactions (28). Using this model, researchers have unraveled important mechanisms and pathways of the human immune response to infective agents and chronic inflammatory diseases. This model involves the intradermal injection of a putative agent (infective or not) and the subsequent generation of a local skin blister (using a vacuum machine). The exudate harvested from the artificial blister offers the opportunity to study the kinetics of the onset and resolution of human inflammation, including the innate and adaptive immune responses to the challenge (29, 30). This review discusses how the skin blister model represents a novel opportunity to understand more about the human response to a common pathogen implicated in the pathogenesis of periodontitis (*P. gingivalis*) and its close links with other systemic inflammatory comorbidities. A brief review of the immune responses implicated in the onset of periodontitis and its systemic consequences is followed by a description of the novel *P. gingivalis* human challenge model, outlining its potential benefits and limitations.

## 2 Host susceptibility to periodontitis and its link to inflammatory comorbidities: the case for immune response

Periodontitis occurs as a result of polymicrobial synergy and dysbiosis in susceptible hosts. A combination of impaired immunity and systemic and environmental factors could be a vital determinant of the shift within the gingival ecosystem to dysbiosis. The local host inflammatory response is responsible for periodontal tissue destruction (31). For the focus of this review, a prominent role will be given to the trait of immunologic susceptibility which not only affects the onset of periodontitis and its progression but also wound-healing potential during various treatment steps of periodontitis (32).

Studies on understanding the immunologic susceptibility to periodontitis have focused on population-level genetic analyses, including single nucleotide polymorphisms (SNPs) of inflammatory genes – reviewed extensively in (10) and (33). Recent genome-wide association studies (GWAS) have contributed to the identification of novel loci encoding immune response-related genes associated with periodontitis (34–39). The genome studies have expanded further using single-cell RNA sequencing (sc-RNA seq), such as in the experiments of Williams et al. demonstrating the presence of exaggerated responsiveness in stromal cells along with augmented neutrophil and leukocytes infiltration in periodontal tissue of patients suffering from periodontitis (40). Further sub-clustering of inflammatory cells, particularly myeloid cells, also exhibited the amplification of inflammatory response. Specifically, macrophages derived from gingival tissue of periodontitis individuals exhibited pro-inflammatory phenotype by highly expressing NLRP3, an inflammasome-mediating IL-1β production (41). Translating the evidence reported by SNP, GWAS, and sc-RNA seq studies ideally requires *in vivo* human evidence on the exact mechanisms of the pathogenesis of periodontitis.

Refined understanding of the cellular and molecular mechanisms of host-microbe interactions in the pathogenesis of periodontitis, as extensively reviewed elsewhere (42–44), has important implications for the treatment of periodontitis. The current approach of standard periodontal treatments incorporates dental biofilm reduction surrounding the teeth and periodontal tissues (non-surgical and surgical periodontal therapies) (45). However, the mechanical interventions for periodontitis have been unable to improve clinical outcomes in certain patients, not to mention those with systemic inflammatory diseases such as diabetes - uncontrolled disease is detrimental to periodontal health and can boost treatment complications (46, 47). Host modulation therapy, therefore, emerges to overcome the limitation of mechanical debridement and has been proposed as an adjunctive therapy alongside the standard treatments (48, 49). Despite the numerous studies of novel host-modulating agents for periodontitis, the majority of the agents are still in preclinical studies and have yet to move forward to clinical applications (50).

Understanding the immunological basis of periodontitis could also be relevant when assessing the links between the disease and other common chronic inflammatory diseases. Periodontitis is not confined to the gingival tissues but has been consistently associated with increased systemic inflammation and altered circulating inflammatory cell profiles (8, 51). Clinical evidence confirms that patients with periodontitis exhibit elevated levels of pro-inflammatory mediators, including IL-1β, IL-6, TNFα, and C-reactive protein (CRP), as well as increased neutrophil counts in peripheral blood when compared to controls (52–55). This relationship seems reversible as effective treatment of periodontitis results in a normalization of some inflammatory biomarkers, including CRP (56, 57). Some cellular markers, however, do not seem to be affected by the resolution of local gingival inflammation as achieved by conventional dental cleaning sessions (13, 14, 58). Dampening the hepatic systemic response due to a reduction of local inflammatory cytokines is the most plausible mechanism explaining a reduction of CRP after treatment of periodontitis. The lack of resolution of immunologic changes could result from a trained immunity state induced by periodontitis-associated systemic inflammation (59). As previously described, this process could be defined as innate immune memory involving changes in the hematopoietic stem and progenitor cells (HSPCs) in the bone marrow after exposure to certain microorganisms (60).

Both clinical and animal experimental evidence support the notion that trained immunity could be a key mechanism explaining the systemic health implications of periodontitis. A recent clinical study confirmed a possible link between raised periodontal inflammation and femoral bone marrow activity in patients with periodontitis compared to controls (61). Ishai and colleagues provided evidence that increased bone marrow activity could mediate the relationship between periodontitis and arterial inflammation (62). Further, two key animal experiments demonstrated HSPC’s involvement when infection with pathogens linked to periodontitis was performed. In mice, continuous release (subcutaneous injection) of a keystone periodontal pathogen, *P. gingivalis*, caused increased osteoclast differentiation in the bone marrow that was IL-6-dependent (63). Indeed, isolated peripheral blood mononuclear cells from patients with periodontitis compared to healthy controls are predisposed to RANKL-induced osteoclastogenesis *ex vivo* (64). Further experimental evidence shows that periodontitis-triggered maladaptive innate immune memory in the bone marrow produces myeloid cells with enhanced inflammatory responses. Li and colleagues further confirmed that either experimental periodontitis or arthritis-induced trained immunity plays a role in the two-way relationship between periodontitis and arthritis (65). In this context, enhanced responses in inflammatory cells (e.g. increased production of pro-inflammatory mediators upon *ex vivo* stimulation) are evident in patients with periodontitis; the immune cells retain these phenotypes following the treatment of periodontitis – extensively discussed in ref (8). This begs the question: could trained immunity be triggered by periodontitis in humans? It remains an important question that warrants further investigation.

## 3 The translational potential of skin blister model in inflammation research

*In vivo* human inflammation models can be classified into two categories based on the delivery route of the challenge. The first group relies upon intravenous injection of bacterial endotoxins (66), cytokine administration (67), typhoid vaccine (68), and strenuous exercise (69), for example. Given the potentially robust systemic inflammatory reaction and possible side effects, most of these models require being conducted in highly controlled research centers. Alternatively, less invasive inflammation models triggered by local intervention have been developed. Our group has previously characterized a human model of treatment of periodontitis (70). A transient systemic inflammatory response of moderate magnitude has been described extensively (70–72). Local administration of inflammatory stimuli, for example, in the skin, has also been proposed as a possible human model to characterize human reaction to infective agents. This localized tissue inflammation model facilitates the investigation of leukocyte migration and accumulation of soluble inflammatory mediators at the site of injection. In a human lung infection model, different encounters (e.g. endotoxin, ozone, and rhinovirus) to induce inflammation have been utilized and can be delivered through inhalation. Sample isolation is needed to understand the temporal inflammatory profile after the challenge. However, it can be more challenging to perform and less comfortable for the participants (sputum collection) (73), limiting its applicability and reliability.

**Figure 1:**
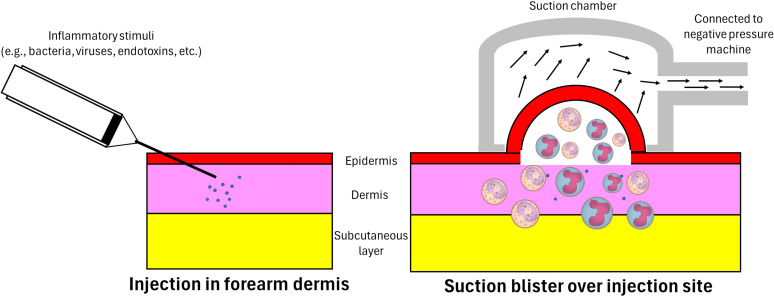
Intradermal injection and inflammatory exudate acquisition. A 30 gauge needle attached to a 1-ml syringe is used to administer the inflammatory stimuli into the dermis just under the epidermis. A suction chamber is then positioned and secured over the injection site at a pre-specified time-point. The negative pressure generated by an electronic vacuum machine separates the epidermis from the dermis and draws in the accumulated inflammatory exudate, including inflammatory cells and soluble mediators – in response to the introduced stimulus – at the dermis. As a result, a unilocular space between the dermis and epidermis containing inflammatory exudate is formed (an artificial blister).

Skin models of inflammation offer instead a contained and practical alternative to these systemic challenge models. Indeed, dermal models are characterized by minimal invasiveness, multiple sites accessible to monitor during the induction of inflammation, and easier access for sample/fluid collection. Several types of skin inflammation models have been established in humans based on the categorization of agents used, including tuberculin purified protein derivative (PPD) (74), BCG (75), cantharidin (28), Candida (candin) antigen (76), varicella-zoster virus (VZV) (77), *Escherichia coli* (*E. coli*) (78), and *Streptococcus pneumoniae* (*S. pneumoniae*) (79) and lipopolysaccharides (LPS) (80). An artificial blister is created at the inoculation site using a vacuum machine secured on the skin at selected time points after intradermal injection. The negative pressure generated by the machine separates the epidermis from the dermis (**Figure 1**), allowing confinement, collection and drainage of an inflammatory exudate (81). The intradermal injection of the pathogen resulted in localized inflammatory responses with complete local clearance of the pathogen and no systemic dissemination of the pathogen (30, 82). As a self-resolving inflammatory model in humans, these experiments have been replicated by several research groups and have shown good tolerability for participants recruited (including healthy individuals of different ages and patients with chronic diseases) (83–85).

For example, in human immunobiology the cantharidin-induced skin blister model helped to identify impaired efferocytosis in the elderly. Furthermore, using this model researchers identified oral intake of losmapimod – a selective p38 mitogen-activated protein kinase (MAPK) inhibitor – as a therapeutic intervention capable of reversing this inflammatory resolution defect (84). A similar model has also been validated to evaluate the anti-inflammatory properties of anti-TNF (adalimumab) and corticosteroids in reducing innate inflammatory cell recruitment to the injection site (86).

Amplified acute inflammation onset with delayed resolution was evaluated in patients with ulcerative colitis when challenged with UV-killed *E. coli* and *S. pneumoniae* prior to suction skin blister formation (83). Using the same model of UV-killed *E. coli* injection, another study reported prolonged immune alterations following the resolution of inflammation, challenging the notion that homeostasis is achieved after acute inflammation resolves (78). Several endogenous specialized pro-resolving mediators (SPMs) and their receptors have been identified as key regulators of the initial phase of inflammation resolution, and furthermore the administration of exogenous SPMs has been shown to boost this process in humans (87).

The skin blister model has also contributed to a greater understanding of the dynamics of adaptive immune cells in humans. When a tuberculin PPD injection (skin blister model) was utilized to study human T cell recall responses, numerous regulatory mechanisms for maintaining human memory T cells *in vivo* were observed (88). The isolation of antigen-specific memory T cells from the suction blister followed by *in vitro* stimulation to become regulatory T cells (Tregs) was attainable, and this was demonstrated to have a suppressing role in inflammation (89). Synchronized production of IL-2 by CD4+ T cells in blister fluid and skin biopsy was showed in the model, enabling the non-invasive characterization of potential defects in adaptive immune response in the elderly. Blister fluid analyses became a valid alternative to skin biopsy to characterize the dynamic of host response to various triggers (74).

A series of further experiments involving patients with active, latent, or cured tuberculosis (TB) was able to describe the role of IL-17A and Th17 responses in patients with active TB, when compared to those with latent TB (85). When TB treatment was administered in these patients, the same immune changes resolved, with a decrease in IL-17A and Th17 responses. In this case, it was skin biopsy samples following the intradermal injection of tuberculin PPD that were analyzed (85). Subsequent experiments using this model enabled the assessment of the safety and specificity of novel monoclonal antibodies aimed at reducing the recruitment of Th17 during adaptive immunity (90). When candin was used as an inflammatory insult, the skin blister model revealed that restricted immunosurveillance by memory T cells in the ageing population is dependent on the reduction of macrophage-derived TNFα production (76). Finally, when using VZV as an insult, researchers showed how regulatory T cells may be derived from memory T cells during localized inflammatory responses (91).

## 4 Intradermal injection of UV-killed *P. gingivalis*: an *in vivo* human skin inflammation model to investigate periodontitis and its link to systemic diseases

### 4.1 Skin inflammation model triggered by *P. gingivalis* in periodontal research

For over three decades, mice studies have utilized a skin inflammation model to assess host response to *P. gingivalis*, a common Gram-negative anaerobe implicated in the development of periodontitis. The most common model is the subcutaneous chamber model, which involves implanting a titanium coil chamber beneath the skin in the dorso-lumbar region of the mouse. After the coil placement has healed, *P. gingivalis* is injected into the chamber, allowing researchers to evaluate the host response to the bacterial challenge (92). When *P. gingivalis* injection was repeated on the model, researchers were able to understand the initiation of adaptive immune response in a time-dependent manner (93, 94). Moreover, studies using this model demonstrated how *P. gingivalis* can evade the host oxidative immune response and survive by escaping host antimicrobial killing (95, 96). Thanks to this model, researchers were able to demonstrate how *P. gingivalis* (through the function of gingipain) stimulated a specific inflammatory response and, at the same time, inhibited the antimicrobial killing and neutrophil phagocytosis resulting in an oral microbial dysbiosis which could ultimately lead to the development of periodontitis (97).

Although skin inflammation models using cantharidin (28), tuberculin PPD (29, 77), varicella zoster virus, *E.* coli (30), or *S. pneumoniae* (79) have been successfully used before, experiments using *P. gingivalis* in humans have not been attempted to date. Cantharidin is a vesicant secreted by blister beetles and 24 hours after its application onto the skin, a blister is formed on the skin and accumulates in the subsequent 72 hours. The 24 hour skin blister represents the acute phase of inflammation while the resolution of inflammation can be observed in the blister 72 hours after dermal cantharidin application (84, 98). When using other inflammatory insults, the induction of an artificial blister on the skin is needed with the help of a small electronic vacuum machine. Specifically, negative pressure generated by this machine is applied over the injection site, generating an artificial blister (**Figure 1**). The timing of blister formation is dependent upon the inflammatory insult and experimental design of the study (29, 30, 77, 79). For example, artificial blister formation was performed 7 days after the injection of either tuberculin PPD or VZV to investigate T-cell recall profiling and responses in humans (29, 77). When using *E. coli* or *S. pneumoniae* it took 4- and 48-hours post-injection to evaluate the acute response and inflammation resolution (30, 79). Host effects when challenging the skin has been previously reported. For example, when challenging participants with UV-killed *E. coli*, moderate swelling and mild discomfort in the axillary region were reported, but receded after 24 hours, with a complete resolution within 48 hours (30). Furthermore, when adopting the artificial blister creation, skin pigmentation has been reported and resolves within 4-6 weeks in Caucasians participants, although it could persist longer in darker skin types (30).

### 4.2 The potential benefits and limitations of the *P. gingivalis*-induced skin inflammation model

Research investigating the immunological mechanisms involved in the pathogenesis and progression of periodontitis and its link to other systemic chronic diseases has primarily used human *ex vivo* or animal *in vivo* models. Whilst on the one hand, these *ex vivo* and animal experiments allowed us to understand the changes that occur within the gingival tissue in response to dental biofilm accumulation and its potential systemic implications, the translation of this evidence into humans remains to be confirmed. A specific human model of inflammation in periodontal research could therefore improve our understanding of how humans respond to challenges of individual or multiple micro-organisms implicated with a common oral disease, as well as how it might impact systemic wellbeing.

Mouse models for periodontitis have been comprehensively reviewed by Rojas and colleagues (99). The current limitation of the model encompasses substantial immunological differences between mice and humans (100). Seok et al. showed that the genomic responses to different inflammatory stimuli in humans poorly correlated with mice models (101). Hence, the translational projection of results - in particular the investigation of the host-microbial interactions in periodontitis – to meet human research frameworks is limited and difficult (99). Moreover, humanized mice models for immune investigations have been developed, but the complications due to xenogeneic transfers are ongoing challenges that researchers need to overcome, not to mention the potential bias of results owing to xenogeneic immune response (102). Therefore, human *in vivo* models could address those issues as experiments, on humans offer the benefits of being relevant to the complexity of human *in vivo* physiology.

Despite the versatility of animal models in biomedical research such as transgenic mice, the successful translation of animal studies for human clinical applications remains questionable. This is particularly true of inflammation-driven disease studies (103). For instance, more than 100 clinical trials have failed to reproduce the successful approach of modulating septic response to infection in animals revealed by sepsis studies (104). Such preclinical research can result in expensive and fruitless clinical implications for patient care. These unreliable findings mean that the overreliance of animals in preclinical research may not contribute to tangible clinical benefit, instead becoming an unnecessary and avoidable research inefficiency (105). Conducting research utilizing human *in vivo* models seems wise to reduce the use of animals in research and that in compliance with the Three Rs principle (replacement, reduction, and refinement) that underlies appropriate involvement of animals in research (humane animal experimental research) (106).

There would be several advantages in developing a human experimental model for *P. gingivalis.* Firstly, it could help compare individuals’ host response to various types of micro-organism, including different genetically modified periodontal bacterial strains (i.e. lacking certain virulence factors such as gingipains and/or pathogen-associated molecular patterns such as LPS). Secondly, there is growing interest in understanding the potential impact of periodontitis on the overall host inflammatory burden and development/progression of other common chronic diseases such as diabetes, Alzheimer’s and cardiovascular diseases. Gathering information on the host response (both cellular and biochemical) to common pathogens linked to periodontitis such as *P. gingivalis* could confirm a number of hypothetical causal pathways/mechanisms that have been proposed to date. Thirdly, performing these experiments in individuals presenting with healthy gingival tissues or already affected by periodontitis could help understand the different susceptibility traits linked to the onset and development of periodontitis. Finally, the same model could provide evidence to support new strategies for addressing periodontal inflammation and managing patients with periodontitis. This could enhance approaches to reducing damage and improving the chances of regaining soft and hard tissues around teeth (107–110). SPMs in periodontitis are extensively discussed elsewhere (111, 112). The exact SPMs that humans produce endogenously are inflammatory stimulus-dependent, meaning that different microorganisms may trigger distinct patterns of SPM production (113, 114). A human inflammation-induced skin model including *P.gingivalis* could provide insights into which SPM signatures drive the resolution of inflammation.

It is also important to note the limitations of using the skin infection model. First, the use of inactivated bacteria in such a model will not necessarily correlate with the same responses expected when challenged by live micro-organisms. Second, several lines of evidence adapting pathogens to induce skin-inflammation (30, 79) highlighted a substantial variation in outcomes between individuals following the challenge, as well as low numbers of cells retrieved from the blister exudates. Third, when using pre-specified time points, a novel skin blister model using periodontal pathogens would be limited to short-lived exposures to micro-organisms, and researchers could only yield two samples per participant (30, 79). Multiple sites on the skin could be used to perform the same challenge (80), but this will inevitably increase the complexity and potential risk of the model for potential participants, without mentioning the increased ethical concerns/considerations (discussed below).

### 4.3 Intradermal injection of UV-killed *P. gingivalis*: practical considerations

Although some limitations were previously discussed, the safety and practical aspects of developing a model of *P. gingivalis* skin blister inflammation in humans must be in a full adherence to ethical standards of medical research involving human participants as detailed in the Declaration of Helsinki (115). In order to achieve that, the model must first involve a team of immunologists, microbiologists and experts in clinical pharmacology confirming that the research utilizing the model is scientifically sound and rigorous in design. Supported by the urgency of the need for human models discussed above, research utilizing the inflammation-induced skin blister human model may only be conducted if the importance of the research outcomes outweighs risks and burdens to the research participants. As the model includes the intradermal injection of *P. gingivalis*, measures to minimize risks of the intervention must be implemented, including deliberate selection of bacterial strains and their growth media and tolerable reactions to the participants after the injection (discussed below). Lastly, appropriate compensation for the volunteers who participate in the studies using the model must be ensured because the procedure of intradermal injection and immune reactions that follow are likely to cause participant’s discomfort and inconvenience.

*P. gingivalis* was selected as putative pathogen relevant to periodontitis and able to generate a local inflammatory response. Indeed, it is a keystone pathogen in periodontitis, and its systemic effects have been reported in both pre-clinical and clinical studies (23, 97, 116, 117). The first task to begin the development of the model includes the selection of which *P. gingivalis* strain and growth media to allow safe but effective intradermal injection. The wild-type strain 2561 could be considered a candidate of *P. gingivalis* rather than either strains BAA-308 (W83) or 53978 (W50), in-keeping with the safety concerns of attempting for the first time the intradermal injection of a Gram-negative micro-organism, hence a less virulent strain could be chosen (118–122). Second, the possible growth media for *P. gingivalis*; in particular, past experiments mainly used brain heart infusion (BHI) to culture the bacteria (23, 123, 124). This media, however, contains bovine brains, and safety concerns were evaluated about the possible risk of cross-contamination during the process of bacterial isolation. Alternative growth media had therefore been evaluated such as PYG broth (125–128). This growth media was chosen to grow the bacterium in anaerobic conditions similar to the BHI media, and it is also free from animal-derived products (128).

The next step in the development of such a model following the establishment of *P. gingivalis* isolates would be to confirm a safe route of administration of *P. gingivalis* for human experimental applications. This could be achieved by generating inactivated, structurally preserved, and measurable micro-organisms. UV exposure is selected as a potent technique to inactivate bacteria without compromising the bacteria’s structural integrity. UV light only targets bacterial DNA, particularly inducing pyrimidine dimers between DNA thymine bases. Subsequently, the micro-organism becomes incapable of replicating without losing their protein structures, including virulence factors (129). A set of experiments could then designed to determine the amount of UV-killed *P. gingivalis* for intradermal injection that is safe and sufficient in generating a self-resolving intradermal inflammatory response. Adequate bacterial quantification experiments should be completed to enable the generation of a sufficient number of micro-organisms to use in the model (130). Viable plate counts in combination with turbidity (optical density) measurements could be used to plot bacterial growth (130, 131) and appropriate quantification of UV-killed *P. gingivalis*. After measurable inactivated *P. gingivalis* is established, dose escalation experiments to determine bacterial numbers that humans could tolerate but sufficient to generate a reproducible inflammatory response, are required. Several lines of evidence can be reviewed and used to complete this part of the experiments using different quantities of UV-killed *P. gingivalis* (30, 79, 83).

## 5 Conclusion

The inflammation-induced skin blister model is a well-established human model of inflammation that successfully captures the immunoinflammatory status of healthy and diseased individuals. Specifically, the model can unveil the complex mechanisms of human immune responses in chronic inflammatory diseases and/or in response to specific inflammatory stimuli/infections. The host laboratory developed for the first time a safe and reproducible UV-killed *P. gingivalis* intradermal injection model followed by artificial suction blister formation and collection of cellular and soluble inflammatory infiltrates.

Periodontal research could use this novel model to enhance our understanding of which immunological mechanisms occur in humans with periodontitis and shed greater light on the potential link between this common chronic disease and other systemic inflammatory disorders.

## References

[1] Lamont RJ Koo H Hajishengallis G. The oral microbiota: dynamic communities and host interactions. Nat Rev Microbiol. (2018) 16:745–59. doi: 10.1038/s41579-018-0089-x PMC6278837 30301974

[2] World Health Organization. Oral Health 2023 . Available online at: https://www.who.int/news-room/fact-sheets/detail/oral-health (accessed November 19, 2023).

[3] Kinane DF Stathopoulou PG Papapanou PN. Periodontal diseases. Nat Rev Dis primers. (2017) 3:1–14. doi: 10.1038/nrdp.2017.38 28805207

[4] Graziani F Music L Bozic D Tsakos G. Is periodontitis and its treatment capable of changing the quality of life of a patient? Br Dental J. (2019) 227:621–5. doi: 10.1038/s41415-019-0735-3 31605074

[5] Botelho J MaChado V Leira Y Proença L Chambrone L Mendes JJ. Economic burden of periodontitis in the United States and Europe: An updated estimation. J Periodontology. (2022) 93:373–9. doi: 10.1002/JPER.21-0111 34053082

[6] Bezerra B Monajemzadeh S Silva D Pirih FQ. Modulating the immune response in periodontitis. Front Dental Med. (2022) 3:879131. doi: 10.3389/fdmed.2022.879131

[7] Genco RJ Sanz M. Clinical and public health implications of periodontal and systemic diseases: An overview. Periodontology 2000. (2020) 83:7–13. doi: 10.1111/prd.12344 32385880

[8] Irwandi RA Kuswandani SO Harden S Marletta D D'Aiuto F. Circulating inflammatory cell profiling and periodontitis: A systematic review and meta-analysis. J Leukocyte Biol. (2022) 111:1069–96. doi: 10.1002/JLB.5RU1021-524R 35199874

[9] Hajishengallis G. Interconnection of periodontal disease and comorbidities: Evidence, mechanisms, and implications. Periodontology 2000. (2022) 89:9–18. doi: 10.1111/prd.12430 35244969 PMC9018559

[10] Laine ML Crielaard W Loos BG. Genetic susceptibility to periodontitis. Periodontology 2000. (2012) 58:37–68. doi: 10.1111/j.1600-0757.2011.00415.x 22133366

[11] Caetano AJ D'Agostino EM Sharpe P Nibali L. Expression of periodontitis susceptibility genes in human gingiva using single-cell RNA sequencing. J Periodontal Res. (2022) 57:1210–8. doi: 10.1111/jre.13057 36170299

[12] Medara N Lenzo JC Walsh KA Reynolds EC O’Brien-Simpson NM Darby IB. Peripheral neutrophil phenotypes during management of periodontitis. J periodontal Res. (2021) 56:58–68. doi: 10.1111/jre.12793 32803891

[13] Radvar M Tavakol AJ Naghib ZBM Naseh M-R Arab H-R. The effect of periodontal treatment on IL-6 production of peripheral blood monocytes in aggressive periodontitis and chronic periodontitis patients. Iran J Immunol. (2008) 5: 100–6. 18523355 10.22034/iji.2008.48559

[14] Ling MR Chapple IL Matthews JB. Peripheral blood neutrophil cytokine hyper-reactivity in chronic periodontitis. Innate Immun. (2015) 21:714–25. doi: 10.1177/1753425915589387 26055820

[15] Tang L Li X Bai Y Wang P Zhao Y. MicroRNA-146a negatively regulates the inflammatory response to Porphyromonas gingivalis in human periodontal ligament fibroblasts via TRAF6/p38 pathway. J periodontology. (2019) 90:391–9. doi: 10.1002/jper.2019.90.issue-4 30378773

[16] Cheng WC van Asten SD Burns LA Evans HG Walter GJ Hashim A. Periodontitis-associated pathogens P. gingivalis and A. actinomycetemcomitans activate human CD14+ monocytes leading to enhanced Th17/IL-17 responses. Eur J Immunol. (2016) 46:2211–21. doi: 10.1002/eji.201545871 PMC5031191 27334899

[17] Ding P-H Darveau RP Wang C-Y Jin L. 3LPS-binding protein and its interactions with P. gingivalis LPS modulate pro-inflammatory response and Toll-like receptor signaling in human oral keratinocytes. PloS One. (2017) 12:e0173223. doi: 10.1371/journal.pone.0173223 28384159 PMC5383028

[18] Khonsuphap P Pavasant P Irwandi RA Leethanakul C Vacharaksa A. Epithelial cells secrete interferon-γ Which suppresses expression of receptor activator of nuclear factor kappa-B ligand in human mandibular osteoblast-like cells. J periodontology. (2017) 88:e65–74. doi: 10.1902/jop.2016.160476 27762732

[19] Irwandi RA Khonsuphap P Limlawan P Vacharaksa A. miR-302a-3p regulates RANKL expression in human mandibular osteoblast-like cells. J Cell Biochem. (2018) 119:4372–81. doi: 10.1002/jcb.v119.6 29058810

[20] Zhao Q Wen J Ouyang X Liu J Liu W Zhang S. Whole-transcriptome analysis of periodontal tissue and construction of immune-related competitive endogenous RNA network. BMC Oral Health. (2022) 22:1–9. doi: 10.1186/s12903-022-02401-0 36045361 PMC9429583

[21] Li W Zhang Z Wang Z-M. Differential immune cell infiltrations between healthy periodontal and chronic periodontitis tissues. BMC Oral Health. (2020) 20:1–10. doi: 10.1186/s12903-020-01287-0 PMC7590666 33109155

[22] Jin R Ning X Liu X Zhao Y Ye G. Porphyromonas gingivalis-induced periodontitis could contribute to cognitive impairment in Sprague–Dawley rats via the P38 MAPK signaling pathway. Front Cell Neurosci. (2023) 17:1141339. doi: 10.3389/fncel.2023.1141339 37056710 PMC10086325

[23] Dominy SS Lynch C Ermini F Benedyk M Marczyk A Konradi A. Porphyromonas gingivalis in Alzheimer’s disease brains: Evidence for disease causation and treatment with small-molecule inhibitors. Sci Adv. (2019) 5:eaau3333. doi: 10.1126/sciadv.aau3333 30746447 PMC6357742

[24] Wang J Zhou Y Ren B Zou L He B Li M. The role of neutrophil extracellular traps in periodontitis. Front Cell infection Microbiol. (2021) 11:639144. doi: 10.3389/fcimb.2021.639144 PMC8012762 33816343

[25] Kim TS Silva LM Theofilou VI Greenwell-Wild T Li L Williams DW. Neutrophil extracellular traps and extracellular histones potentiate IL-17 inflammation in periodontitis. J Exp Med. (2023) 220 (9):e20221751. doi: 10.1084/jem.20221751 37261457 PMC10236943

[26] Kenny EF Herzig A Krüger R Muth A Mondal S Thompson PR. Diverse stimuli engage different neutrophil extracellular trap pathways. Elife. (2017) 6:e24437. doi: 10.7554/eLife.24437 28574339 PMC5496738

[27] Farrera C Fadeel B. Macrophage clearance of neutrophil extracellular traps is a silent process. J Immunol. (2013) 191:2647–56. doi: 10.4049/jimmunol.1300436 23904163

[28] Jenner W Motwani M Veighey K Newson J Audzevich T Nicolaou A. Characterisation of leukocytes in a human skin blister model of acute inflammation and resolution. PloS One. (2014) 9:e89375. doi: 10.1371/journal.pone.0089375 24603711 PMC3945731

[29] Holm LL Vukmanovic-Stejic M Blauenfeldt T Benfield T Andersen P Akbar AN. A suction blister protocol to study human T-cell recall responses *in vivo* . JoVE (Journal Visualized Experiments). (2018) 138:e57554. doi: 10.3791/57554-v PMC6126709 30148487

[30] Motwani MP Flint JD De Maeyer RP Fullerton JN Smith AM Marks DJ. Novel translational model of resolving inflammation triggered by UV-killed E. coli. J Pathology: Clin Res. (2016) 2:154–65. doi: 10.1002/cjp2.v2.3 PMC4958736 27499924

[31] Hajishengallis G. Immunomicrobial pathogenesis of periodontitis: keystones, pathobionts, and host response. Trends Immunol. (2014) 35:3–11. doi: 10.1016/j.it.2013.09.001 24269668 PMC3947349

[32] Sima C Glogauer M. Neutrophil dysfunction and host susceptibility to periodontal inflammation: current state of knowledge. Curr Oral Health Rep. (2014) 1:95–103. doi: 10.1007/s40496-014-0015-x

[33] Sayad A Gholami L Mirzajani S Omrani MD Ghafouri-Fard S Taheri M. Genetic susceptibility for periodontitis with special focus on immune-related genes: A concise review. Gene Rep. (2020) 21:100814. doi: 10.1016/j.genrep.2020.100814

[34] Divaris K Monda KL North KE Olshan AF Reynolds LM Hsueh W-C. Exploring the genetic basis of chronic periodontitis: a genome-wide association study. Hum Mol Genet. (2013) 22:2312–24. doi: 10.1093/hmg/ddt065 PMC3652417 23459936

[35] Rhodin K Divaris K North K Barros S Moss K Beck J. Chronic periodontitis genome-wide association studies: gene-centric and gene set enrichment analyses. J Dental Res. (2014) 93:882–90. doi: 10.1177/0022034514544506 PMC4213253 25056994

[36] Shaffer JR Polk DE Wang X Feingold E Weeks DE Lee M-K. Genome-wide association study of periodontal health measured by probing depth in adults ages 18– 49 years. G3: Genes Genomes Genet. (2014) 4:307–14. doi: 10.1534/g3.113.008755 PMC3931564 24347629

[37] Sanders A Sofer T Wong Q Kerr K Agler C Shaffer J. Chronic periodontitis genome-wide association study in the Hispanic Community Health Study/Study of Latinos. J Dental Res. (2017) 96:64–72. doi: 10.1177/0022034516664509 PMC5347427 27601451

[38] Munz M Richter GM Loos BG Jepsen S Divaris K Offenbacher S. Meta-analysis of genome-wide association studies of aggressive and chronic periodontitis identifies two novel risk loci. Eur J Hum Genet. (2019) 27:102–13. doi: 10.1038/s41431-018-0265-5 PMC6303247 30218097

[39] Shungin D Haworth S Divaris K Agler CS Kamatani Y Keun Lee M. Genome-wide analysis of dental caries and periodontitis combining clinical and self-reported data. Nat Commun. (2019) 10:2773. doi: 10.1038/s41467-019-10630-1 31235808 PMC6591304

[40] Williams DW Greenwell-Wild T Brenchley L Dutzan N Overmiller A Sawaya AP. Human oral mucosa cell atlas reveals a stromal-neutrophil axis regulating tissue immunity. Cell. (2021) 184:4090–104. e15. doi: 10.1016/j.cell.2021.05.013 34129837 PMC8359928

[41] Qian S-J Huang Q-R Chen R-Y Mo J-J Zhou L-Y Zhao Y. Single-cell RNA sequencing identifies new inflammation-promoting cell subsets in Asian patients with chronic periodontitis. Front Immunol. (2021) 12:711337. doi: 10.3389/fimmu.2021.711337 34566966 PMC8455889

[42] Kinane DF Preshaw PM Loos BG Periodontology WGotSEWo. Host-response: understanding the cellular and molecular mechanisms of host-microbial interactions–consensus of the Seventh European Workshop on Periodontology. J Clin periodontology. (2011) 38:44–8. doi: 10.1111/j.1600-051X.2010.01682.x 21323703

[43] Meyle J Chapple I. Molecular aspects of the pathogenesis of periodontitis. Periodontology 2000. (2015) 69:7–17. doi: 10.1111/prd.2015.69.issue-1 26252398

[44] Hajishengallis G Korostoff JM. Revisiting the Page & Schroeder model: the good, the bad and the unknowns in the periodontal host response 40 years later. Periodontology 2000. (2017) 75:116–51. doi: 10.1111/prd.2017.75.issue-1 PMC5539911 28758305

[45] Sanz M Herrera D Kebschull M Chapple I Jepsen S Berglundh T. Treatment of stage I–III periodontitis—The EFP S3 level clinical practice guideline. J Clin periodontology. (2020) 47:4–60. doi: 10.1111/jcpe.v47.s22 PMC7891343 32383274

[46] Preshaw PM Bissett SM. Periodontitis and diabetes. Br Dental J. (2019) 227:577–84. doi: 10.1038/s41415-019-0794-5 31605062

[47] Nibali L Gkranias N Mainas G Di Pino A. Periodontitis and implant complications in diabetes. Periodontology 2000. (2022) 90:88–105. doi: 10.1111/prd.12451 35913467 PMC9805043

[48] Giannobile WV. Host-response therapeutics for periodontal diseases. J periodontology. (2008) 79:1592–600. doi: 10.1902/jop.2008.080174 PMC2583215 18673015

[49] Hajishengallis G Chavakis T Lambris JD. Current understanding of periodontal disease pathogenesis and targets for host-modulation therapy. Periodontology 2000. (2020) 84:14–34. doi: 10.1111/prd.12331 32844416 PMC7457922

[50] Preshaw PM. Host modulation therapy with anti-inflammatory agents. Periodontology 2000. (2018) 76:131–49. doi: 10.1111/prd.2018.76.issue-1 29193331

[51] Hajishengallis G Chavakis T. Local and systemic mechanisms linking periodontal disease and inflammatory comorbidities. Nat Rev Immunol. (2021) 21:426–40. doi: 10.1038/s41577-020-00488-6 PMC7841384 33510490

[52] Moutsopoulos NM Madianos PN. Low-grade inflammation in chronic infectious diseases: paradigm of periodontal infections. Ann New York Acad Sci. (2006) 1088:251–64. doi: 10.1196/annals.1366.032 17192571

[53] Górska R Gregorek H Kowalski J Laskus-Perendyk A Syczewska M Madaliński K. Relationship between clinical parameters and cytokine profiles in inflamed gingival tissue and serum samples from patients with chronic periodontitis. J Clin periodontology. (2003) 30:1046–52. doi: 10.1046/j.0303-6979.2003.00425.x 15002890

[54] Loos BG Craandijk J Hoek FJ Dillen P van der Velden U. Elevation of systemic markers related to cardiovascular diseases in the peripheral blood of periodontitis patients. J periodontology. (2000) 71:1528–34. doi: 10.1902/jop.2000.71.10.1528 11063384

[55] Botelho J MaChado V Hussain SB Zehra SA Proença L Orlandi M. Periodontitis and circulating blood cell profiles: a systematic review and meta-analysis. Exp Hematology. (2021) 93:1–13. doi: 10.1016/j.exphem.2020.10.001 33068648

[56] MaChado V Botelho J Escalda C Hussain SB Luthra S Mascarenhas P. Serum C-reactive protein and periodontitis: a systematic review and meta-analysis. Front Immunol. (2021) 3054. doi: 10.3389/fimmu.2021.706432 PMC8355591 34394107

[57] Luthra S Orlandi M Hussain SB Leira Y Botelho J MaChado V. Treatment of periodontitis and C-reactive protein: A systematic review and meta-analysis of randomized clinical trials. J Clin Periodontology. (2023) 50:45–60. doi: 10.1111/jcpe.13709 PMC10087558 35946825

[58] Sharma S Sridhar S McIntosh A Messow C-M Aguilera EM Del Pinto R. Periodontal therapy and treatment of hypertension-alternative to the pharmacological approach. A systematic review and meta-analysis. Pharmacol Res. (2021) 166:105511. doi: 10.1016/j.phrs.2021.105511 33617973

[59] Irwandi RA Chiesa ST Hajishengallis G Papayannopoulos V Deanfield JE D’Aiuto F. The roles of neutrophils linking periodontitis and atherosclerotic cardiovascular diseases. Front Immunol. (2022) 13:915081. doi: 10.3389/fimmu.2022.915081 35874771 PMC9300828

[60] Netea MG Domínguez-Andrés J Barreiro LB Chavakis T Divangahi M Fuchs E. Defining trained immunity and its role in health and disease. Nat Rev Immunol. (2020) 20:375–88. doi: 10.1038/s41577-020-0285-6 PMC7186935 32132681

[61] Noz MP Plachokova AS Smeets EM Aarntzen EH Bekkering S Vart P. An explorative study on monocyte reprogramming in the context of periodontitis *in vitro* and *in vivo* . Front Immunol. (2021) 12:695227. doi: 10.3389/fimmu.2021.695227 34484192 PMC8414567

[62] Ishai A Osborne MT El Kholy K Takx RA Ali A Yuan N. Periodontal disease associates with arterial inflammation via potentiation of a hematopoietic-arterial axis. JACC: Cardiovasc Imaging. (2019) 12:2271–3. doi: 10.1016/j.jcmg.2019.05.015 PMC6932860 31326471

[63] Zhao Y Li Z Su L Ballesteros-Tato A Katz J Michalek SM. Frontline science: Characterization and regulation of osteoclast precursors following chronic Porphyromonas gingivalis infection. J Leucocyte Biol. (2020) 108:1037–50. doi: 10.1002/JLB.1HI0620-230R 33463750

[64] Herrera BS Bastos AS Coimbra LS Teixeira SA Rossa J Van Dyke TE. Peripheral blood mononuclear phagocytes from patients with chronic periodontitis are primed for osteoclast formation. J periodontology. (2014) 85:e72–81. doi: 10.1902/jop.2013.130280 24059638

[65] Li X Wang H Yu X Saha G Kalafati L Ioannidis C. Maladaptive innate immune training of myelopoiesis links inflammatory comorbidities. Cell. (2022) 185:1709–27. e18. doi: 10.1016/j.cell.2022.03.043 35483374 PMC9106933

[66] Michie HR Manogue KR Spriggs DR Revhaug A O'Dwyer S Dinarello CA. Detection of circulating tumor necrosis factor after endotoxin administration. New Engl J Med. (1988) 318:1481–6. doi: 10.1056/NEJM198806093182301 2835680

[67] Rosenberg SA Lotze MT Muul LM Leitman S Chang AE Ettinghausen SE. Observations on the systemic administration of autologous lymphokine-activated killer cells and recombinant interleukin-2 to patients with metastatic cancer. New Engl J Med. (1985) 313:1485–92. doi: 10.1056/NEJM198512053132327 3903508

[68] Feasey NA Levine MM. Typhoid vaccine development with a human challenge model. Lancet. (2017) 390:2419–21. doi: 10.1016/S0140-6736(17)32407-8 28965714

[69] Suzuki K Nakaji S Yamada M Totsuka M Sato K Sugawara K. Systemic inflammatory response to exhaustive exercise. Cytokine kinetics. Exercise Immunol review. (2002) 8:6–48. 12690937

[70] D’Aiuto F Parkar M Andreou G Suvan J Brett PM Ready D. Periodontitis and systemic inflammation: control of the local infection is associated with a reduction in serum inflammatory markers. J Dental Res. (2004) 83:156–60. 10.1177/154405910408300214 14742655

[71] D’aiuto F Nibali L Parkar M Suvan J Tonetti M. Short-term effects of intensive periodontal therapy on serum inflammatory markers and cholesterol. J Dental Res. (2005) 84:269–73. doi: 10.1177/154405910508400312 15723869

[72] D'Aiuto F Nibali L Mohamed-Ali V Vallance P Tonetti M. Periodontal therapy: A novel non-drug-induced experimental model to study human inflammation. J Periodontal Res. (2004) 39:294–9. doi: 10.1111/j.1600-0765.2004.00741.x 15324349

[73] van der Merwe R Molfino NA. Challenge models to assess new therapies in chronic obstructive pulmonary disease. Int J Chronic Obstructive Pulmonary Dis. (2012) 7:597–605. doi: 10.2147/COPD.S30664 PMC3459659 23055710

[74] Akbar A Reed J Lacy K Jackson S Vukmanovic-Stejic M Rustin M. Investigation of the cutaneous response to recall antigen in humans. Clin Exp Immunol. (2013) 173(2):163–72. doi: 10.1111/cei.12107 PMC3722916 23607634

[75] Minassian AM Satti I Poulton ID Meyer J Hill AV McShane H. A Human Challenge Model for Mycobacterium tuberculosis Using M ycobacterium bovis Bacille Calmette-Guérin. J Infect Diseases. (2012) 205:1035–42. doi: 10.1093/infdis/jis012 PMC3295601 22396610

[76] Agius E Lacy KE Vukmanovic-Stejic M Jagger AL Papageorgiou A-P Hall S. Decreased TNF-α synthesis by macrophages restricts cutaneous immunosurveillance by memory CD4+ T cells during aging. J Exp Med. (2009) 206:1929–40. doi: 10.1084/jem.20090896 PMC2737169 19667063

[77] Patel NP Vukmanovic-Stejic M Suarez-Farinas M Chambers ES Sandhu D Fuentes-Duculan J. Impact of zostavax vaccination on T-cell accumulation and cutaneous gene expression in the skin of older humans after varicella zoster virus antigen–specific challenge. J Infect Dis. (2018) 218:S88–98. doi: 10.1093/infdis/jiy420 PMC6151076 30247603

[78] Motwani MP Newson J Kwong S Richard-Loendt A Colas R Dalli J. Prolonged immune alteration following resolution of acute inflammation in humans. PloS One. (2017) 12:e0186964. doi: 10.1371/journal.pone.0186964 29073216 PMC5658111

[79] Szylar G Wysoczanski R Marshall H Marks DJ José R Ehrenstein MR. A novel Streptococcus pneumoniae human challenge model demonstrates Treg lymphocyte recruitment to the infection site. Sci Rep. (2022) 12:3990. doi: 10.1038/s41598-022-07914-w 35256717 PMC8901783

[80] Buters TP Hameeteman PW Jansen IM van Hindevoort FC Ten Voorde W Florencia E. Intradermal lipopolysaccharide challenge as an acute *in vivo* inflammatory model in healthy volunteers. Br J Clin Pharmacol. (2022) 88:680–90. doi: 10.1111/bcp.14999 PMC9290695 34293819

[81] Smith TJ Wilson MA Young AJ Montain SJ. A suction blister model reliably assesses skin barrier restoration and immune response. J Immunol Methods. (2015) 417:124–30. doi: 10.1016/j.jim.2015.01.002 25585263

[82] Collins GB de Souza Carvalho J Jayasinghe SC Gumuliauskaite U Lowe DM Thomas DC. A new model measuring bacterial phagocytosis and phagolysosomal oxidation in humans using the intradermal injection of methylene blue–labeled Escherichia coli. J Leukocyte Biol. (2024), qiae217. doi: 10.1093/jleuko/qiae217 39412158 PMC11879004

[83] Wysoczanski R Kendall AC Motwani M Vega R Rahman FZ McCartney S. Ulcerative colitis is characterized by amplified acute inflammation with delayed resolution. bioRxiv. (2019), 870139. doi: 10.1101/870139

[84] De Maeyer RP van de Merwe RC Louie R Bracken OV Devine OP Goldstein DR. Blocking elevated p38 MAPK restores efferocytosis and inflammatory resolution in the elderly. Nat Immunol. (2020) 21:615–25. doi: 10.1038/s41590-020-0646-0 PMC7983074 32251403

[85] Pollara G Turner CT Rosenheim J Chandran A Bell LC Khan A. Exaggerated IL-17A activity in human *in vivo* recall responses discriminates active tuberculosis from latent infection and cured disease. Sci Trans Med. (2021) 13:eabg7673. doi: 10.1126/scitranslmed.abg7673 PMC7610803 33952677

[86] Dinh PHD Corraza F Mestdagh K Kassengera Z Doyen V Michel O. Validation of the cantharidin-induced skin blister as an *in vivo* model of inflammation. Br J Clin Pharmacol. (2011) 72:912–20. doi: 10.1111/j.1365-2125.2011.04020.x PMC3244638 21595743

[87] Motwani MP Colas RA George MJ Flint JD Dalli J Richard-Loendt A. Pro-resolving mediators promote resolution in a human skin model of UV-killed Escherichia coli–driven acute inflammation. JCI Insight. (2018) 3 (6):e94463. doi: 10.1172/jci.insight.94463 29563331 PMC5926908

[88] Reed JR Vukmanovic-Stejic M Fletcher JM Soares MVD Cook JE Orteu CH. Telomere erosion in memory T cells induced by telomerase inhibition at the site of antigenic challenge *in vivo* . J Exp Med. (2004) 199:1433–43. doi: 10.1084/jem.20040178 PMC2211820 15148341

[89] Vukmanovic-Stejic M Sandhu D Sobande TO Agius E Lacy KE Riddell N. Varicella zoster–specific CD4+ Foxp3+ T cells accumulate after cutaneous antigen challenge in humans. J Immunol. (2013) 190:977–86. doi: 10.4049/jimmunol.1201331 PMC3552094 23284056

[90] Bouma G Zamuner S Hicks K Want A Oliveira J Choudhury A. CCL20 neutralization by a monoclonal antibody in healthy subjects selectively inhibits recruitment of CCR6+ cells in an experimental suction blister. Br J Clin Pharmacol. (2017) 83:1976–90. doi: 10.1111/bcp.13286 PMC5555862 28295451

[91] Vukmanovic-Stejic M Agius E Booth N Dunne PJ Lacy KE Reed JR. The kinetics of CD4+ Foxp3+ T cell accumulation during a human cutaneous antigen-specific memory response *in vivo* . J Clin Invest. (2008) 118:3639–50. doi: 10.1172/JCI35834 PMC2556297 18924611

[92] Genco CA Cutler CW Kapczynski D Maloney K Arnold RR. A novel mouse model to study the virulence of and host response to Porphyromonas (Bacteroides) gingivalis. Infection immunity. (1991) 59:1255–63. doi: 10.1128/iai.59.4.1255-1263.1991 PMC257836 2004807

[93] Houri-haddad Y Soskolne W Halabi A Barak V Shapira L. Repeat bacterial challenge in a subcutaneous chamber model results in augmented tumour necrosis factor-α and interferon-γ response, and suppression of interleukin-10. Immunology. (2000) 99:215–20. doi: 10.1046/j.1365-2567.2000.00965.x PMC2327143 10692039

[94] Houri-Haddad Y Soskolne WA Shapira L. Immunization to Porphyromonas gingivalis enhances the local pro-inflammatory response to subcutaneous bacterial challenge. J Clin Periodontology. (2001) 28:476–82. doi: 10.1034/j.1600-051x.2001.028005476.x 11350512

[95] Mydel P Takahashi Y Yumoto H Sztukowska M Kubica M Gibson FC III. Roles of the host oxidative immune response and bacterial antioxidant rubrerythrin during Porphyromonas gingivalis infection. PloS pathogens. (2006) 2:e76. doi: 10.1371/journal.ppat.0020076 16895445 PMC1522038

[96] Burns E Eliyahu T Uematsu S Akira S Nussbaum G. TLR2-dependent inflammatory response to Porphyromonas gingivalis is MyD88 independent, whereas MyD88 is required to clear infection. J Immunol. (2010) 184:1455–62. doi: 10.4049/jimmunol.0900378 20042569

[97] Maekawa T Krauss JL Abe T Jotwani R Triantafilou M Triantafilou K. Porphyromonas gingivalis manipulates complement and TLR signaling to uncouple bacterial clearance from inflammation and promote dysbiosis. Cell Host Microbe. (2014) 15:768–78. doi: 10.1016/j.chom.2014.05.012 PMC4071223 24922578

[98] Jenner WJ Gilroy DW. Assessment of leukocyte trafficking in humans using the cantharidin blister model. JRSM Cardiovasc disease. (2012) 1:1–5. doi: 10.1258/cvd.2012.012009 PMC3738319 24175059

[99] Rojas C García MP Polanco AF González-Osuna L Sierra-Cristancho A Melgar-Rodríguez S. Humanized mouse models for the study of periodontitis: an opportunity to elucidate unresolved aspects of its immunopathogenesis and analyze new immunotherapeutic strategies. Front Immunol. (2021) 12:663328. doi: 10.3389/fimmu.2021.663328 34220811 PMC8248545

[100] Mestas J Hughes CC. Of mice and not men: differences between mouse and human immunology. J Immunol. (2004) 172:2731–8. doi: 10.4049/jimmunol.172.5.2731 14978070

[101] Seok J Warren HS Cuenca AG Mindrinos MN Baker HV Xu W. Genomic responses in mouse models poorly mimic human inflammatory diseases. Proc Natl Acad Sci. (2013) 110:3507–12. doi: 10.1073/pnas.1222878110 PMC3587220 23401516

[102] Shultz LD Brehm MA Garcia-Martinez JV Greiner DL. Humanized mice for immune system investigation: progress, promise and challenges. Nat Rev Immunol. (2012) 12:786–98. doi: 10.1038/nri3311 PMC3749872 23059428

[103] Marshall LJ Bailey J Cassotta M Herrmann K Pistollato F. Poor translatability of biomedical research using animals—A narrative review. Alternatives to Lab Animals. (2023) 51:102–35. doi: 10.1177/02611929231157756 36883244

[104] Marshall JC. Why have clinical trials in sepsis failed? Trends Mol Med. (2014) 20:195–203. doi: 10.1016/j.molmed.2014.01.007 24581450

[105] Pound P Bracken MB. Is animal research sufficiently evidence based to be a cornerstone of biomedical research? Bmj. (2014) 348:g3387. doi: 10.1136/bmj.g3387 24879816

[106] Russell WMS Burch RL Hume CW. The principles of humane experimental technique. London: Methuen (1959).

[107] Lee C-T Teles R Kantarci A Chen T McCafferty J Starr JR. Resolvin E1 reverses experimental periodontitis and dysbiosis. J Immunol. (2016) 197:2796–806. doi: 10.4049/jimmunol.1600859 PMC5026932 27543615

[108] Abdalla HB Puhl L Rivas CA Wu Y-C Rojas P Trindade-da-Silva CA. Modulating the sEH/EETs axis restrains specialized proresolving mediator impairment and regulates T cell imbalance in experimental periodontitis. J Immunol. (2024) 212:433–45. doi: 10.4049/jimmunol.2300650 PMC10866374 38117781

[109] Albuquerque-Souza E Schulte F Chen T Hardt M Hasturk H Van Dyke TE. Maresin-1 and resolvin E1 promote regenerative properties of periodontal ligament stem cells under inflammatory conditions. Front Immunol. (2020) 11:585530. doi: 10.3389/fimmu.2020.585530 33101318 PMC7546375

[110] Zarrough AE Hasturk H Stephens DN Van Dyke TE Kantarci A. Resolvin D1 modulates periodontal ligament fibroblast function. J Periodontology. (2023) 94:683–93. doi: 10.1002/JPER.22-0462 PMC10354588 36416879

[111] Van Dyke TE. Pro-resolving mediators in the regulation of periodontal disease. Mol aspects Med. (2017) 58:21–36. doi: 10.1016/j.mam.2017.04.006 28483532 PMC5660638

[112] Van Dyke TE. Shifting the paradigm from inhibitors of inflammation to resolvers of inflammation in periodontitis. J periodontology. (2020) 91:S19–25. doi: 10.1002/JPER.20-0088 PMC8142079 32441774

[113] Basil MC Levy BD. Specialized pro-resolving mediators: endogenous regulators of infection and inflammation. Nat Rev Immunol. (2016) 16:51–67. doi: 10.1038/nri.2015.4 26688348 PMC5242505

[114] Lee C-T Li R Zhu L Tribble GD Zheng WJ Ferguson B. Subgingival microbiome and specialized pro-resolving lipid mediator pathway profiles are correlated in periodontal inflammation. Front Immunol. (2021) 12:691216. doi: 10.3389/fimmu.2021.691216 34177951 PMC8222734

[115] Association WM. World Medical Association Declaration of Helsinki: ethical principles for medical research involving human participants. JAMA. (2024), E1–E4. doi: 10.1001/jama.2024.21972 39425955

[116] Hajishengallis G Liang S Payne MA Hashim A Jotwani R Eskan MA. Low-abundance biofilm species orchestrates inflammatory periodontal disease through the commensal microbiota and complement. Cell Host Microbe. (2011) 10:497–506. doi: 10.1016/j.chom.2011.10.006 22036469 PMC3221781

[117] Mougeot JC Stevens C Paster B Brennan M Lockhart P Mougeot FB. Porphyromonas gingivalis is the most abundant species detected in coronary and femoral arteries. J Oral Microbiol. (2017) 9:1281562. doi: 10.1080/20002297.2017.1281562 28326156 PMC5328378

[118] Igboin CO Griffen AL Leys EJ. Porphyromonas gingivalis strain diversity. J Clin Microbiol. (2009) 47:3073–81. doi: 10.1128/JCM.00569-09 PMC2756895 19675220

[119] Baker P Dixon M Evans R Roopenian D. Heterogeneity of Porphyromonas gingivalis strains in the induction of alveolar bone loss in mice. Oral Microbiol Immunol. (2000) 15:27–32. doi: 10.1034/j.1399-302x.2000.150105.x 11155161

[120] Wilensky A Gabet Y Yumoto H Houri-Haddad Y Shapira L. Three-dimensional quantification of alveolar bone loss in Porphyromonas gingivalis-infected mice using micro-computed tomography. J periodontology. (2005) 76:1282–6. doi: 10.1902/jop.2005.76.8.1282 16101359

[121] Chen T Hosogi Y Nishikawa K Abbey K Fleischmann RD Walling J. Comparative whole-genome analysis of virulent and avirulent strains of Porphyromonas gingivalis. J bacteriology. (2004) 186:5473–9. doi: 10.1128/JB.186.16.5473-5479.2004 PMC490943 15292149

[122] Seers CA Mahmud ASM Huq NL Cross KJ Reynolds EC. Porphyromonas gingivalis laboratory strains and clinical isolates exhibit different distribution of cell surface and secreted gingipains. J Oral Microbiol. (2021) 13:1858001. doi: 10.1080/20002297.2020.1858001 PMC7733959 33391630

[123] Xie M Tang Q Yu S Sun J Mei F Zhao J. Porphyromonas gingivalis disrupts vascular endothelial homeostasis in a TLR-NF-κB axis dependent manner. Int J Oral Science. (2020) 12:28. doi: 10.1038/s41368-020-00096-z PMC7527479 32999278

[124] Liu Y Wu Z Nakanishi Y Ni J Hayashi Y Takayama F. Infection of microglia with Porphyromonas gingivalis promotes cell migration and an inflammatory response through the gingipain-mediated activation of protease-activated receptor-2 in mice. Sci Rep. (2017) 7:11759. doi: 10.1038/s41598-017-12173-1 28924232 PMC5603557

[125] Biedermann A Kriebel K Kreikemeyer B Lang H. Interactions of anaerobic bacteria with dental stem cells: an *in vitro* study. PloS One. (2014) 9:e110616. doi: 10.1371/journal.pone.0110616 25369260 PMC4219685

[126] Ebbers M Lübcke PM Volzke J Kriebel K Hieke C Engelmann R. Interplay between P. gingivalis, F. nucleatum and A. actinomycetemcomitans in murine alveolar bone loss, arthritis onset and progression. Sci Rep. (2018) 8:15129. doi: 10.1038/s41598-018-33129-z 30310087 PMC6181973

[127] Kriebel K Hieke C Engelmann R Potempa J Müller-Hilke B Lang H. Porphyromonas gingivalis peptidyl arginine deiminase can modulate neutrophil activity via infection of human dental stem cells. J innate Immun. (2018) 10:264–78. doi: 10.1159/000489020 PMC6158109 29860256

[128] Versalovic J. Manual of clinical microbiology. Washington, DC: American Society for Microbiology Press (2011).

[129] Reed NG. The history of ultraviolet germicidal irradiation for air disinfection. Public Health Rep. (2010) 125:15–27. doi: 10.1177/003335491012500105 PMC2789813 20402193

[130] Van Alst AJ LeVeque RM Martin N DiRita VJ. Growth curves: generating growth curves using colony forming units and optical density measurements. MyJoVE Corporation. (2016).

[131] Maier RM Pepper IL. Bacterial growth. In: Environmental microbiology. Cambridge, Massachusetts: Elsevier (2015). p. 37–56.

